# Persons with Epilepsy: Between Social Inclusion and Marginalisation

**DOI:** 10.1155/2016/2018509

**Published:** 2016-04-26

**Authors:** Simona Mlinar, Davorina Petek, Živa Cotič, Metka Mencin Čeplak, Marjan Zaletel

**Affiliations:** ^1^Faculty of Medicine, University of Ljubljana, Vrazov Trg 2, SI-1104 Ljubljana, Slovenia; ^2^Faculty of Medicine, Department of Family Medicine, University of Ljubljana, Poljanski Nasip 58, SI-1000 Ljubljana, Slovenia; ^3^Imperial College London, School of Public Health, Department of Primary Care and Public Health, Global eHealth Unit, Reynolds Building, St. Dunstan's Road, London W6 8RP, UK; ^4^Faculty of Social Sciences, Department of Sociology, University of Ljubljana, Kardeljeva Ploščad 5, SI-1000 Ljubljana, Slovenia; ^5^Department for Vascular Neurology, University Clinical Centre of Ljubljana, Zaloška Cesta 2, SI-1000 Ljubljana, Slovenia

## Abstract

*Background*. Epilepsy is a chronic neurological disorder that can lead to complex psychosocial consequences. Epilepsy can change the social status of persons with epilepsy (PWE) and has an effect on their social inclusion as well as their perception of social inclusion. This study aims to explore subjective experiences with social inclusion of PWE in Slovenia.* Methods*. This study takes a qualitative approach. Eleven semistructured interviews were conducted with eleven participants. Interviews were analysed using thematic analysis.* Results*. Epilepsy has physical, emotional, and social consequences. Physical consequences of epilepsy are mainly tiredness and exhaustion following an epileptic episode, frequently accompanied by headaches. Emotional consequences are different forms of fear. The main social consequence identified is a negative effect on PWE's social network, which leads to (self-)isolation and social distrust.* Conclusion*. PWE experience of social inclusion depends on various psychosocial factors and differs from person to person. The consequences of epilepsy are shown in PWE social contacts and their sense of social inclusion and autonomy.

## 1. Introduction

Epilepsy is a complex disorder. There are several types of epilepsy, which have different causes and symptoms and require different treatments. This study considers all types of epilepsy and does not distinguish between its specific types. Epilepsy results in neurological consequences, physical consequences, and psychosocial consequences. One of psychosocial consequences of epilepsy is a change in social inclusion of persons with epilepsy (PWE) [1]. Social inclusion has several connotations. It can mean participation, capacity development, and societal welfare increase [2], as well as capacity to be actively included in society and contribute to society in economic, social, psychological, and political sense [3]. Social inclusion can help explain the consequences of social stratification, principles of societal order, and placement of persons in the centre of society or its margins [4]. Various definitions of social inclusion stem from the ideal of inclusive society. In an inclusive society everyone feels valued, differences are respected, needs are met, and living with dignity is the norm [5]. This study defines social inclusion as PWE's active inclusion in society.

Epileptic episodes are sudden and frequently dangerous and carry increased risk of injury, hospitalisation, and death. Insufficient knowledge of epilepsy and inappropriate first aid can result in inappropriate treatment, physical disability, and social exclusion [6]. PWE are at a high risk of developing mental disorders, such as anxiety and depression. They frequently experience social issues and have trouble finding a partner. PWE can have lower levels of education and higher unemployment rates [7] and are less likely to participate in social activities [8, 9].

Social inclusion is an important element of quality of life for PWE. Recent research has shown that stigma experienced by PWE contributes to increased levels of psychopathology, decrease in social contact and social capital, and lower quality of life [10]. Concealing and disclosing epilepsy have an important role in managing stigma experienced by PWE. As a strategy of stigma control, concealing epilepsy has its weaknesses. Failure in hiding epilepsy can lead to increased stigmatisation. This process leads into a cycle of secrecy, societal detachment and isolation, and antisocial behavior [9]. Morgan and colleagues have found strong correlation between prevalence of epilepsy and social deprivation [11].

Past research has focused on individual aspects of epilepsy, namely, on its influence on the quality of life of PWE in relation to stigmatisation, discrimination, employment opportunities, psychiatric epilepsy occurrence, style of living, burden of epilepsy, and pregnancy [12–14]. These factors have a significant effect on PWE quality of life, which is lower in comparison to the general population [15–17]. These factors are important aspects of PWE care [18]. As far as we are aware, no studies aiming to explore subjective aspects of PWE social inclusion and effects of subjective experiences of epilepsy on social inclusion have been conducted.

Discrimination and stigmatisation of PWE are social determinants of health with poor social prognosis [19, 20]. Both affect PWE more than epileptic episodes [21]. It is important to explore and understand PWE experience with social inclusion. Objective indicators of social inclusion, such as social network size, level of education, employment status and subjective experiences of inclusion/exclusion, social contacts, and relationships, need to be explored [4].

Qualitative approach to research was chosen based on our research aims and objectives. Qualitative research encompasses research methods describing and explaining participant experiences, behavior, relationships, and social environment [22, 23], such as how PWE experience epilepsy and the effect of PWE experience of social inclusion.

The aim of this study is to explore subjective experience of PWE social inclusion in Slovenia. The research objectives of this study were to determine how PWE experience epilepsy and the effect of PWE experience of social inclusion. We used qualitative methods to explore how selected aspects of epilepsy affect the life of PWE and how PWE participate in a society and contribute to it.

The National Medical Ethics Committee of the Republic of Slovenia approved the protocol for this study in February 2012 (doc. number 109/01/12).

## 2. Methods

### 2.1. Theoretical Framework and Research Approach

We have chosen phenomenology as the theoretical framework for this study. Phenomenology takes into consideration experience and how people understand experience as a primary source of realisation. Phenomenology explores the structure of conscious subjective experience from the first-person point of view [24]. We have chosen phenomenology because it is an appropriate framework for exploring social dimension of health or specific disorders, such as epilepsy. The objective of phenomenology is to answer “how is it to have a particular experience” [25]. Phenomenology as a theoretical framework is intended to precisely describe, analyse, and interpret experience from an individual point of view and is expressed as a personal story [26, 27]. Its aim is to uncover the meaning of intensity of being, to develop a deeper understanding and a detailed identification of personal experience.

This is a qualitative study. We used semistructured interviews to collect stories about epilepsy. Interview questions were developed with the intention to motivate participant to tell stories about their experience of epilepsy: they were used to steer participants towards the main theme of the study.

### 2.2. Participants

Inclusion criteria were

- subjectively expressed epilepsy diagnosis,
- age of majority,
- legal capacity,
- willingness to tell a story regarding experience with epilepsy.

We included only voluntary participants. Participants were recruited using* snowball sampling* technique. We sent e-mail invitations containing relevant information on the study and provisional questions on story structure to various addresses collected by the research team. Addressees were asked to forward the invitation to PWE or other persons who might know PWE. When PWE contacted us themselves, we invited them for an interview.

Eleven participants participated in semistructured interviews. At the time of the interview, all participants were taking medication for epilepsy. Participants signed a written consent form on voluntary participation in this study. Their consent could be revoked at any time during the study. Participant anonymity was achieved through coding of their personal details.

### 2.3. Interviews

The interviews took place between November 2011 and March 2012 at various locations in Slovenia. Participants determined where the interview would take place. Five interviews took place at PWE's homes, three interviews in a public space (i.e., café), and three interviews at the League Against Epilepsy Society headquarters.

Basic demographic data was collected from participants (see Table 1). During the interviews, participants were encouraged to spontaneously talk about epilepsy. Participants were permitted to emphasise events of personal importance. Interviews were administered by one person (SM) and taped. The shortest interview took 35 minutes and the longest interview took 130 minutes.

### 2.4. Analysis

Interviews were transcribed word-for-word into Microsoft Word documents. The transcripts were sent to participants for authorisation. After receiving authorisation, transcripts were analysed and interpreted using content analysis. Content analysis aims to achieve exhaustive and broad description of a certain phenomenon. Additionally, content analysis is used when studying sensitive phenomena. It also enables reduction of large parts of text into smaller content categories [28]. We used inductive method for content analysis [29]. Transcripts were coded. We considered codes words or phrases that are directly extracted from text and may represent textual interpretation. Initially, we postulated a unit of analysis as a single interview and a coding unit as a smaller category of content (e.g., a paragraph describing a specific experience or an event). Two individuals (SM and DP) coded the text independently of each other and reached an agreement on the content of the codes. We piloted three interviews to determine the initial codes. We collected codes (with interview citations) in Microsoft Excel and used them to determine categories and subcategories.

We merged similar codes in superior and subordinate categories, which we then used to form a coding frame. This represented the final phase of data analysis.

During the interpretation phase, we initially transferred one coding unit to analysis unit. Each interview text was examined to determine the context in which the code appears. The code was then substantiated by the original citation. Contextual and interpretative analyses were the last phase of the analysis.

Study findings in relation to preliminary research and established concepts were debated among all the members of the research group.

## 3. Results

### 3.1. PWE Sociodemographic Data

The PWE sample in this study consisted of 8 women and 3 men aged between 27 and 64 years (average 43.8 years). Average participant age at the time of their first epileptic episode was 16.8 years. Participants reported the elapsed time since last episode. The shortest amount of time was 2 days. The longest amount of time was 12 years. One of the participants was unable to recall the time of the last epileptic episode.

At the time of the study, three participants were married, one had a partner, and seven were single. Three participants lived alone, one lived with a partner, and seven participants lived with family. Educational levels among the participants were relatively low. Four participants reported finishing primary school, six participants finished secondary school, and one was educated at the university level. Only two participants were unemployed at the time of the study. Three participants were receiving disability pension and one participant was receiving a monthly allowance. Five participants were employed. Three out of eleven participants were members of the League Against Epilepsy Society.

### 3.2. Coding Frames

Epilepsy experience encompasses various dimensions of life. We have focused on four particular areas. These areas were chosen as coding frames due to the significant effect apportioned to them by the participants in their stories.

The decision on the coding frame was based on Flick and Elo and Kyngäs' work describing coding frames as “concept driven” (literature review) and “data-driven” (research data): coding frames are frequently based on both literature and data [29, 30]. 

Therefore, when we construct the coding frame, we took into consideration

- research aims and objectives,
- review of the literature,
- data gathered from the first three interviews.

This approach to constructing coding frames is additionally supported by theoretical guidelines for qualitative studies [28].

We used research data to develop abstract concepts (categories and subcategories, not to be mistaken for themes in the context of thematic analysis). Themes were used because based on their impact on understanding PWE social inclusion and include aspect of physical environment, PWE interaction among themselves and their relations, PWE feelings, and span of the disease. Categories that could contribute to our research question were translated to abstract concept-category forms (e.g., social network and social position), while subcategories encompassed thematically narrower concepts.

Two coding frames were prepared for analysis purpose. The first coding frame refers to the “characteristics and consequences of epilepsy” (see supplementary Appendix  1, in Supplementary Material available online at http://dx.doi.org/10.1155/2016/2018509), while the second coding frame refers to the “social contacts and relationships” PWE (see supplementary Appendix  2).

“The characteristics and consequences of epilepsy” frame encompasses two categories: concealing and disclosing the epilepsy and disease consequence. “The PWE social contacts and relationships” frame encompasses epilepsy PWE experience and social network (see Table 2).

### 3.3. Epilepsy Characteristics and Consequences

Epilepsy is a complex disease. Consequences of epilepsy are not only physical, but also emotional and social. We discuss all three aspects in this chapter.

#### 3.3.1. Physical Consequences

Participants described the physical draining effect of an epileptic episode. Some of them are able to recognize the beginning of an episode, while some of them are not. After an episode, they feel tired and drained, frequently suffer from headaches, and are in need of rest.

*You feel completely without strength [after an attack] and just want to hug someone. Even I find it difficult to understand, even now I don't understand, how is it possible, after an attack, to feel so powerless. (SP)*

During an epileptic episode, six participants suffered minor physical injuries, such as bumps and cuts. One participant sustained major physical injury, which resulted in finger amputation. Four participants did not mention any injuries related to an epileptic episode.

All participants emphasised the importance of recognizing physical response in various circumstances. They all developed ways of recognizing physical signs that could lead to an epileptic episode. Participants explained that they consider the ability to recognize these signs a mean of controlling their epilepsy. Participants reported that they do not participate in actions or avoid circumstances that could lead to an episode and deterioration of their health.

*It was difficult, giving up sport. You can't lie in life and it's similar with this disease. If I said, that I'm taking medication and drink [alcohol] at the same time it wouldn't be possible to expect the treatment to get better. (KS)*

Participants are aware of their physical and psychological limitations. This awareness enables them to better control epilepsy.

#### 3.3.2. Emotional Consequences

Fear was a common consequence of epilepsy emphasised by the participants. They mentioned different forms of fear: fear related to reaction of their friends and family and others, fear that the epilepsy is going to get worse, fear of motherhood, fear of epilepsy heredity, and fear of epilepsy unpredictability. Fear is the main reason for uncertainty. Participants see a correlation between unpredictability of epilepsy and uncertain future. They reported intense feelings of distress upon familiarising themselves with epilepsy, as well as fear of future episodes. They emphasised distress escalation upon familiarising with epilepsy and fear of the next episode.

*I'm afraid because I don't know when the next episode is going to happen and I worry that the disease is going to get worse, sometimes I fear going down the street alone. (SP)*

Participants listed self-confinement and gradual social isolation as consequences of epilepsy. All participants explained that when they experienced an epileptic episode, people who were present did not recognize physical symptoms of epilepsy; the epileptic episode scared them and they reacted in panic.

#### 3.3.3. Social Consequences of Epilepsy

One of the participants explained that epilepsy disclosure affected her social network and made it weaker. Participants perceive social isolation as a worst-case scenario.

*My youngest daughter got mad at me, asked me what was wrong, since I don't socialise with people anymore. She doesn't know what to do with me, I don't know how to laugh anymore. She knows that I always liked company, we used to laugh a lot, I had a lot of friends, but not anymore. Now, I withdraw into myself. Somehow, I don't feel like going out. I don't know why. (ZVS) *

*This caused me to lose a lot of my friends. As I've already told you, they think you are crazy or not completely there. They didn't take it as a disease. They took it as something scary, a taboo. (DJ) *

Two participants reported having a strong social network and a wide circle of friends, despite epilepsy. Nine participants listed epilepsy as a reason for a weak social network and feelings of loneliness. People who find out about epilepsy are sometimes unable to accept it. Consequently, participants experience rejection of contact. Only immediate family members accept epilepsy. Although the majority of participants found support within their families, one participant described the negative impact of her mother not accepting the fact that he has epilepsy. Another participant described an authoritarian relationship with her partner that also affects their children.

*You feel hurt. Lonely. You feel like you're alone in this world. People don't understand, you can't be there, because if you're there and tell them something, they drive you away. I know now, after my friends have distanced themselves, that my family is all there is. (KJ)*

According to participants, limited ability to work and trouble finding employment are reasons for inadequate social inclusion. Eight participants reported epilepsy as the main reason for not being employed. Three participants did not experience any difficulties in finding employment. One participant emphasised that social conventions regarding job performance are high and that that makes epilepsy a difficult factor to control. Participants also emphasised that, during a job interview, they frequently felt that a potential employer was afraid to employ them due to potential liability. One participant felt discriminated against by her colleagues at work and felt that this contributed to her epilepsy.

*When you are whole, you can do anything. You can go to work, drive a car, all doors in life are open to you. There's nothing written about your disease or limitations. When epilepsy is on your record, all doors close. (KJ)*

Epilepsy also has consequences for family or friends living with PWE. All participants explained that knowing that their immediate relatives have to adapt to their disability causes them a lot of worry and represents a significant burden.

*I can't go anywhere alone and this is why I can't relax. I'm constantly under pressure. I think of what I'm doing so they [family members] will be okay. I'm very troubled, that I'm to blame for everything that they [family members] have to give up and accommodate me. (ZVŠ) *

Participants explained that epilepsy has a negative effect on finding a partner. They emphasised that partners fear epilepsy heredity. Seven participants do not have a partner. Five of these participants have previous experience with partnerships breaking down due to epilepsy. Participants believe that epilepsy represents an obstacle to finding a partner and starting a family.

*I find it difficult to tell them [girls] that I have epilepsy, because I was afraid that they will reject me. I understand that if you don't know enough, you can't respond. But, some girls, when I told them that I'm fine, understood, while some others told me that they don't want a patient for a boyfriend immediately. (SL)*

*I had a lot of girlfriends, but always, when I told them that I have epilepsy, it ended. They thought it was hereditary. (IZ)*

One participant emphasised that epilepsy influences self-perception and opens up opportunities for empathy towards other PWE. This participant expressed a wish for a partner with health concerns, because he believes that they could understand each other better.

### 3.4. Managing Epilepsy Information in Social Contacts and Relationships

In this chapter, we listed codes related to participant control of epilepsy disclosure, epilepsy information dissemination, management of information, participant epilepsy experience, and participant experience related to their actions, emotions, well-being, and autonomy.

#### 3.4.1. Strategies

PWE have little control over epilepsy disclosure due to the unpredictable nature of epileptic episodes. Control over epilepsy disclosure can be very important, namely, because of fear of rejection. All participants emphasised the fact that the term epilepsy and sudden disclosure (i.e., epileptic episode) prompt fear among the general population. Although participants cannot fully control epilepsy disclosure, they try to hide it whenever possible. One of the participants developed a way of hiding an episode when it occurs in public.

*When I had company and felt that I will have an episode, I started rummaging through my bag, and by being active I hid the tremors. In my case, except for my family and perhaps a small circle of friends, nobody knew that I have epilepsy. (MR)*

Participants believe that people fear epilepsy because they are not familiar with it and do not recognize its symptoms. Participants would like to have control over epilepsy disclosure. Five participants explained that they do not have control over information circulation, since epilepsy can only be kept private until a public episode occurs. Two participants explained that they can control disclosure, while others did not attach particular importance to it.

Participants explained that they are in control of disclosure (with the exception of unplanned public episode). They emphasised two main causes for disclosure: (i) longer and close relationship with a partner or a friend that leads to disclosure and (ii) disclosure to enable appropriate help. All participants explained that they prefer to personally explain the characteristics of epilepsy in person. This is because they believe that they will contribute to managing stigma associated with epilepsy.

*Because those that know what epilepsy is, just take a step back… I would rather explain in person. Even when I explain in person, I can say that some girls, that I would like to be with and are very nice, just go away because of my disease. (SL)*

All participants emphasised the distress and fear of epilepsy disclosure, especially to potential future partners. They also mentioned fear of consequences of nondisclosure.

*I told a friend who was afraid of it [epilepsy]. She asked me whether it is contagious and if she can get ill. Afterwards I didn't see her for a while, because she was somewhat afraid of me. (PF)*

*The more honest and spontaneous you are, less people resent you and step towards you. (MP)*

Participants reported the dilemma associated with disclosing a diagnosis of epilepsy to a potential employer. Six participants have disclosed having epilepsy during a job interview. Five of those had a negative experience. They are convinced that epilepsy is the reason why they did not get employment. Three participants concealed epilepsy. Two participants have never been employed.

*I decided to find a job. I was sending job applications around. I didn't mention the fact that I have epilepsy. I went for an interview and didn't mention epilepsy. I know, however, that we had to fill out a questionnaire somewhere and we had to write about this epilepsy and I wrote it, but didn't get a job, although I had a chance to get it. (SL)*

Participants had different experiences regarding epilepsy disclosure when it was controlled (own decision to disclose) or when it was not (public episode). Seven participants regretted disclosing the epilepsy irrespective of the manner of disclosure. Four participants were positively surprised that they received support and felt relieved that they did not need to conceal the epilepsy anymore.

#### 3.4.2. Experience

Participants adapt to the epilepsy in different manners. A low feeling of self-worth was identified as one of the main factors in the participants' having trouble trusting others. All participants, except one, have experienced others fearing their epilepsy. Participants believe that fear is a result of epilepsy manifestation—epileptic seizure. Epilepsy symptoms are not always recognizable or are manifested in a frightening manner. Participants emphasised their feelings of hurt resulting from other people's response to epilepsy.

* I had it [epileptic episode] in a discotheque. People almost treaded on me until someone carried me out and laid me down on cold, wet pavement in the middle of November. My sister told people who were outside to call an ambulance. They told her that they won't call an ambulance for someone on drugs. It was terrible, and I felt terrible afterwards. (KJ)*

All participants emphasised the distress they have been experiencing because of epilepsy. They expressed feelings of powerlessness, desperation and insecurity, distress over other people's reaction, loneliness, self-confinement, disassociation, fear of epilepsy deterioration, dependence on others, need for help, anxiety, distress due to unfulfilled expectations, shame, and feeling different and inferior.

*My boyfriend and I broke up six weeks ago, but I took it really well. In the end, I thought it was a miracle that there are people out there who would even look at people like us. (MP)*

Participants feel guilty because they have to rely on others. Their guilt is accompanied by despair and feeling burdened. Their immediate relatives share these feelings. Feelings of powerlessness are also present, because epilepsy cannot yet be cured. All participants have explained that their life has changed completely when they got epilepsy. They had to change their daily activities and plans for the future.

* If we talked like this from the beginning, I would have said things differently, but now I have twenty, twenty five years of life with epilepsy behind be, so I find it difficult to say what the main leap was… Somehow you make it a part of your life… The only thing I want to say is that crises appear as a result of your life-style, that you don't walk the path that is expected of you. (MP)*

Participants are aware of their dependence on the help of others. This correlates with the loss of autonomy in everyday life. Four participants that are married or have a partner have mentioned that their partner or other family members take a paternalistic approach to their relationship. Partner or family member dominance is expressed as concealing information regarding the epilepsy, exceeding concern for PWE or loss of self-determination. Seven participants explained that epilepsy led to a loss of autonomy. Participants believe that the loss of autonomy is related to their need for help after an episode and feeling dependent on others.

## 4. Discussion

Managing epilepsy and its burden are related to knowing one's body and its responses. We were surprised at how detailed participant's descriptions of physical responses in different circumstances were. Participants are highly aware of, and recognize, potential episode triggers. However, episodes still come as a surprise causing insecurity and resulting in distress.

Based on our interviews, the burden of epilepsy can be divided into three groups: physical burden, social burden, and emotional burden. Participants who have not had an episode in a while have less negative emotions, while those who have episodes more frequently relate their physical distress with their epilepsy. Chaplin and colleagues confirm this by linking psychosocial effects of epilepsy with epileptic episode frequency [31].

Recognizing physical signs that precede an episode and potential episode triggers are two main conditions for at least partial and/or temporary management of social environment. Epilepsy is accompanied by prejudice and stigma. This is why PWE attach high significance to managing epilepsy information. Because they expected a negative reaction from their surroundings, our study participants have, whenever possible, tried to conceal epilepsy. Alternatively, they tried to decide to whom and when they will disclose epilepsy. Participants are aware of the unpredictability of epilepsy. Because episodes cannot be predicted, PWE are constantly faced with anxiety. Even those, who have not had an episode in years, experience anxiety. The burden of unpredictability of epilepsy lies in the knowledge that epilepsy cannot be fully controlled. A small change in lifestyle or a spontaneous reaction to an external stimulus can trigger an epileptic episode. Thus, it is not surprising that the experience of an episode plays a central part in participant's stories. Participants are aware that an epileptic episode triggers fear in others. Typical epileptic episode stereotypes, such as cramps, foaming at the mouth, and jerking, accompanied by various sounds, do not contribute to appropriate reactions of those present at the time of an episode. Participants reported that people reacted to the episode inappropriately (putting different object into PWE's mouth) or did not offer help at all. These experiences negatively affect PWE expectations and lowered their trust. Participants began avoiding those who have shown that they do not accept their condition. Our findings correspond to Baker's research on psychosocial burden of epilepsy. Baker found that isolation and withdrawal are common among PWE. Both withdrawal and isolation are consequences of anxiety due to adverse reactions to public epileptic episodes [32].

The strategy of avoiding unpleasant social consequences employed by participants of our study does not decrease distress. This is due to limited control over epilepsy. Interestingly, participants reported that epilepsy disclosure frequently brought them relief. Tröster explains that PWE decide to disclose epilepsy, when they fear that their social counterpart will notice their condition by himself or will be told of it by others [33].

Based on our findings, we can infer that controlling information on epilepsy, concealing or disclosing epilepsy, and epilepsy consequences are closely connected with the feeling of social inclusion. PWE social security depends on successful social inclusion (e.g., employment). Jennum and colleagues have found that epilepsy has a significant socioeconomic influence [34]. Employment is one of the main social security factors. PWE often have lower level of education, are employed on less paid positions, or are unemployed. According to our research participants, disclosing epilepsy to an employer is one of the most significant social relationship dilemmas. The reason for this dilemma is not stigmatisation, but rather fear of liability on the part of the employer. Jacoby and colleagues have found that the main obstacle to PWE employment is the fear of employing PWE. They also note that PWE fear that they will be discriminated and stigmatised against in relation to employment [35]. Those participants who were employed at the time of our study perceive epilepsy as a factor that influences their job performance. However, they are used to epilepsy and have successfully incorporated it into their work. Trust between PWE and their social environment can be established, provided that it is based on mutual acceptance and epilepsy awareness. Trust can be hard to establish if PWE experience other people's rejection.

Epilepsy affects formation of intimate relationships and their quality. Participants feel that epilepsy results in troubles finding a partner or they blame epilepsy for their “single status.” When forming intimate relationships, participants fear a negative response when disclosing epilepsy. Participants are convinced that no one wants a partner who represents a burden. Espínola-Nadurille and colleagues have found similar responses when PWE experienced an end of a relationship with a partner or immediate relatives due to epilepsy or when partner or relatives were present during an episode [20]. Our study shows that the expectation that a disclosure in person might be better for a relationship is not always fulfilled. Those participants that have disclosed epilepsy in person have experienced rejection. Immediate relatives have mostly expressed their rejection indirectly, for example, as a doubt that they will not be able to appropriately respond to an episode.

Participants underlined another significant effect of epilepsy: unwanted loss of autonomy and dependence on others. Both loss of autonomy and dependence are psychosocial burdens. Loss of autonomy can represent a burden for two reasons: PWE understand that the epilepsy is a burden for their immediate relatives and they are not completely independent in everyday tasks. PWE frequently rely on help from others, especially during an episode. Dependence means that PWE involuntarily lose their autonomy. If dependence results in a paternalistic and rigid attitude of others, this leads to permanent loss of autonomy. Excessive care or concern of those close to PWE can be damaging, since they effectively take away PWE's independence. For those PWE who have frequent episodes, independence is an unachievable goal. This is mainly because they are physically unable to take care of themselves after an attack. Feelings of guilt related to their dependence on others are always accompanied by a feeling of powerlessness: PWE feel unable to change their situation. Our study showed that even affection and understanding of other people did not change these feelings. When researching stories of patients with chronic disease, Öhman and colleagues showed similar findings. Chronic patients felt that loss of autonomy influences their daily lives. When trying to meet their basic needs, they were obliged to ask for help [36].

Our study confirms the supposition that epilepsy influences social inclusion of PWE. Epilepsy consequences are reflected in social contacts and relationships that PWE form and in their feelings of social acceptance and autonomy. Epilepsy affects not only PWE but also their families and their whole community. De Boer and colleagues have also reached the same conclusion. This is why the effect of epilepsy on social contacts and relationships is also its key burden. The burden of epilepsy is represented by social exclusion of PWE, which is a result of negative responses of others. Epilepsy is accompanied by stigma [37]. Stigmatisation is a social obstacle for PWE. They face stigmatisation in all aspects of life (education, employment, partnership and forming a family, motherhood, social contact, etc.). The role of parents, siblings, and other close relatives that PWE live or socialise with is of immense importance in the context of social inclusion, especially for establishing relationships based on trust. PWE mostly participate in activities within their family circle. We have found that feelings of acceptance result in strengthening participant endeavours to incorporate epilepsy into their life. Participants who feel accepted by their environment are more involved in it, participate in more pastime activities, and spend more time with a broader circle of people outside of their family and they believe that living with epilepsy does not represent greater difficulties.

One of the strategies employed by our participants to actively influence the attitude of their social environment towards epilepsy is their endeavour to explain to others what type of epilepsy they have, how epilepsy expresses itself, and the feelings they have towards it. Controlling information regarding epilepsy is not limited to concealing epilepsy and avoiding potentially uncomfortable social situations. More importance is attached to actively deal with prejudice, fear, and lack of knowledge. Participants are aware that they can actively combat prejudices accompanying epilepsy and affect the opinion their social counterparts will form of epilepsy and PWE. Hopkins states that PWE have to be their own public relationship officer, who decides what and how much to say and what to conceal [38]. PWE have the ability to positively affect prejudice and associated stigma on a broader social level. Bagič and colleagues have done a study in Croatia where PWE used social marketing to combat prejudice and epilepsy stigmatisation. The results of a follow-up study have shown a high level of tolerance towards PWE and a positive attitude towards children with epilepsy. Based on these results, researchers have concluded that social marketing can be beneficial to positively affecting public attitude towards PWE [39]. However, it is important to note that these findings are not generalizable in regard to positive behavioral attitude towards PWE. We can conclude that PWE are subject to discrimination and stigmatisation.

## 5. Study Limitations

This study has several limitations. We included participants irrespective of their exact diagnosis that is form of epilepsy. Some forms of epilepsy are more difficult to manage, have severe consequences, and carry a different burden. This is why our findings are not generalizable to the entire population of PWE in Slovenia. We also included a relatively small number of participants. We encouraged PWE to describe their experiences independently. However, this was not always possible due to cognitive impairments or additional health issues (besides epilepsy). We might have introduced interviewer bias into analysed stories. Another limitation is the choice of the location of the interview (cafés), which was left to each individual participant due to potential privacy issues related to interview questions. While we did not ask PWE why they chose cafés as meeting places, they often explained that they felt they could not discuss private matters at home in the presence of their relatives. The different length of the interviews might also be considered a limitation since it functions as a subjective factor. However, the nature of semistructured interviews enables participants to discuss a specific question in their own time as freely as possible. The criterion we used to ensure homogeneity was that all participants had to answer all interview questions.

## 6. Conclusion

Through history, epilepsy has attracted prejudice, stereotype, and stigma, factors that go beyond the medical frame of epilepsy. These factors function as barriers towards establishing social contacts and relationships, weaken PWE social networks, and force PWE to control personal information. These factors lead to social isolation, the most radical effect of social exclusion. Changing prejudice, stereotypes, and stigma is a process which involves all of society and takes a lot of time. Social inclusion aims to achieve inclusive society, which is in turn necessary for social inclusion. Inclusive society does not “come to be” by itself but rather forms by means of societal participation. PWE's experience of social inclusion depends on various psychosocial factors and personal perspective.

Awareness of epilepsy needs to be raised. Firstly, PWE need to be informed, so they will be able to address the consequences of epilepsy. Secondly, PWE's family and friends have to be informed of epilepsy to be able to share in the life of PWE, understand them, and react appropriately when or if epilepsy deteriorates. Last but not least, broader society needs to be aware of epilepsy, since their attitude influences how PWE experience epilepsy and its consequences. Media campaigns and other campaigns that aim to raise awareness of epilepsy have proven to be relatively effective, even more so if they included PWE. In Slovenia, targeted campaigns aiming to raise awareness regarding PWE social inclusion are few. There is a lack of research on PWE social inclusion and PWE subjective evaluation of social inclusion. We call for more research in this area, namely, qualitative studies that could explore these themes in depth and recommend PWE involvement in campaigns for epilepsy awareness raising.

## Supplementary Material

Appendix 1 indicates the placement of categories “Concealing/disclosing epilepsy” and “Epilepsy consequences (disease burden)” within the broader coding frame.
Appendix 2 indicates the placement of categories “PWE experience and social network” within the broader coding frame.

## Figures and Tables

**Table 1:** PWE sociodemographic data.

PWE	Age	Marital status	Living arrangements	Level of education	Employment	Age of first symptoms (1st episode)	Time lapsing since last episode	Member of “Društvo liga proti epilepsiji Slovenije”/League against Epilepsy Society
M.SL.I	27	Single	With parents	Secondary school	Employed	13	2 days	No
Z.KJ.I	31	Partner	With partner	Secondary school	Employed	16	5 years	No
Z.MP.I	39	Single	Alone	Secondary school	Employed	19	3 days	No
Z.MR.I	40	Single	Alone	University	Employed	6	6 years	No
M.IZ.I	59	Single	with sister's family	Primary school	Disability pension	18	2 weeks	No
Z.ZVS.I	50	Married	With family	Primary school	Unemployed	22	2 days	No
Z.SP.I	40	Married	With family	Secondary school	Disability pension	14	5 days	No
Z.DJ.I	51	Married	With family	Secondary school	Disability pension	28	1 month	No
M.KS.I	50	Single	Alone	Secondary school	Unemployed	7	12 years	Yes
Z.MF.I	64	Single	With sister	Primary school	Monthly allowance	13	Does not recall	Yes
Z.PF.I	31	Single	With father	Primary school	Employed	29	4 months	Yes

**Table 2:** Themes and codes.

Frame	Categories	Codes
Characteristics and consequences of epilepsy	Concealing/disclosing epilepsy	Hiding/concealing epilepsy, uncontrolled epilepsy disclosure, concealing an episode, controlling information, controlling information disclosure, limited circle of persons aware of the epilepsy, confidentiality, and startling one's environment
	Epilepsy consequences	Physical: losing control over one's body, frightening symptoms, epilepsy unpredictability, difficult epilepsy management, and character change Emotional: epilepsy as punishment, fear of epilepsy heredity, emotional distress, distress escalation related to familiarisation with the epilepsy, loss of control, fear of an episode, concern, insecurity, distrust as defensive mechanism, feeling deprived, and concern due to epilepsy unpredictability Social: barrier to starting a family, partnership idiosyncrasy, distress due to opinions of others, concern regarding motherhood, influence of the epilepsy on finding a partner, self-confinement, concern of friends and family, and family recognition of the burden of epilepsy

PWE social contacts and relationships	PWE experience and social network	Epilepsy disclosure related remorse, information disclosure related relief, positive experience of disclosure, negative experience of disclosure, hiding the epilepsy due to previous negative experience, disclosure distress, fear of consequences related to epilepsy concealment, and wish to disclose
