# Supplementary material for: Persons with Epilepsy: Between Social Inclusion and Marginalisation
Source: Behav Neurol. 2016 Apr 26;2016:2018509. doi: 10.1155/2016/2018509 (PMC4861793; doi:10.1155/2016/2018509)

Appendix 1: Epilepsy characteristics and consequences

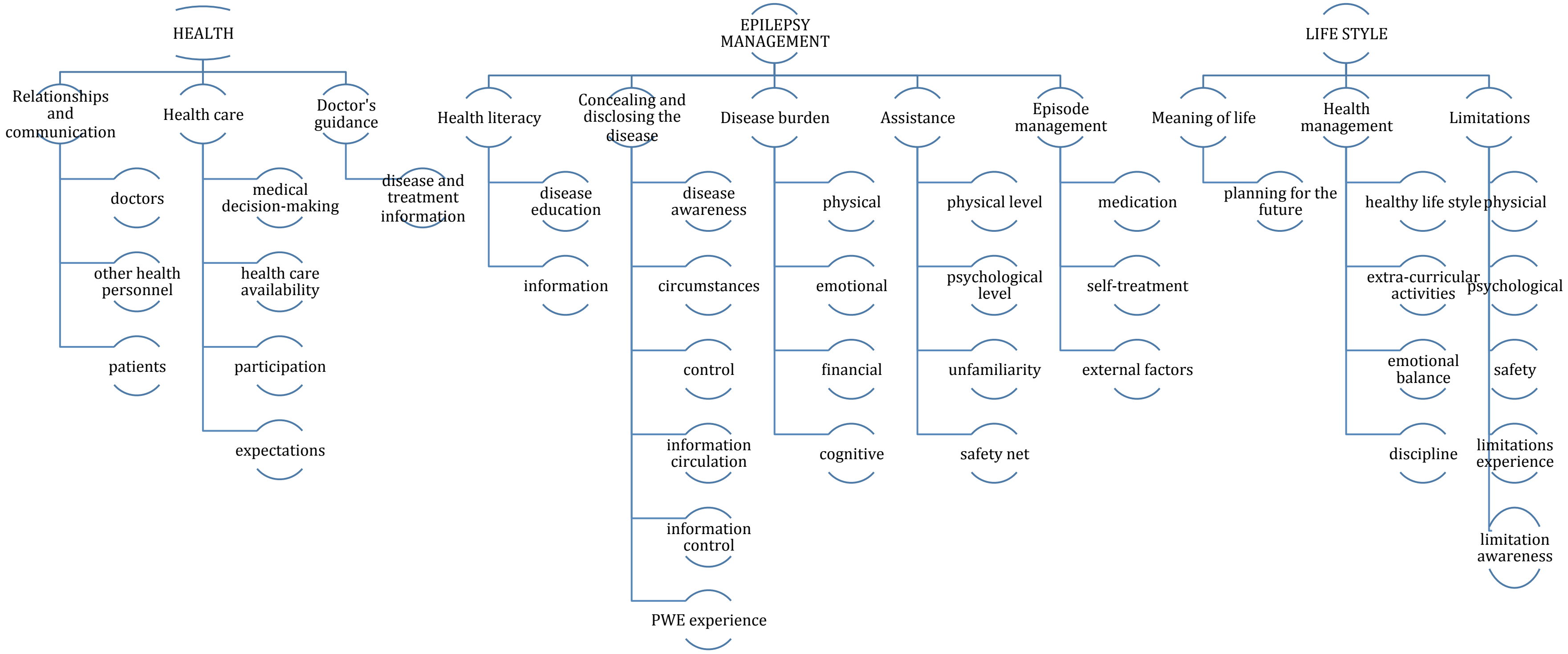

Appendix 2: PWE Social contacts and relationships

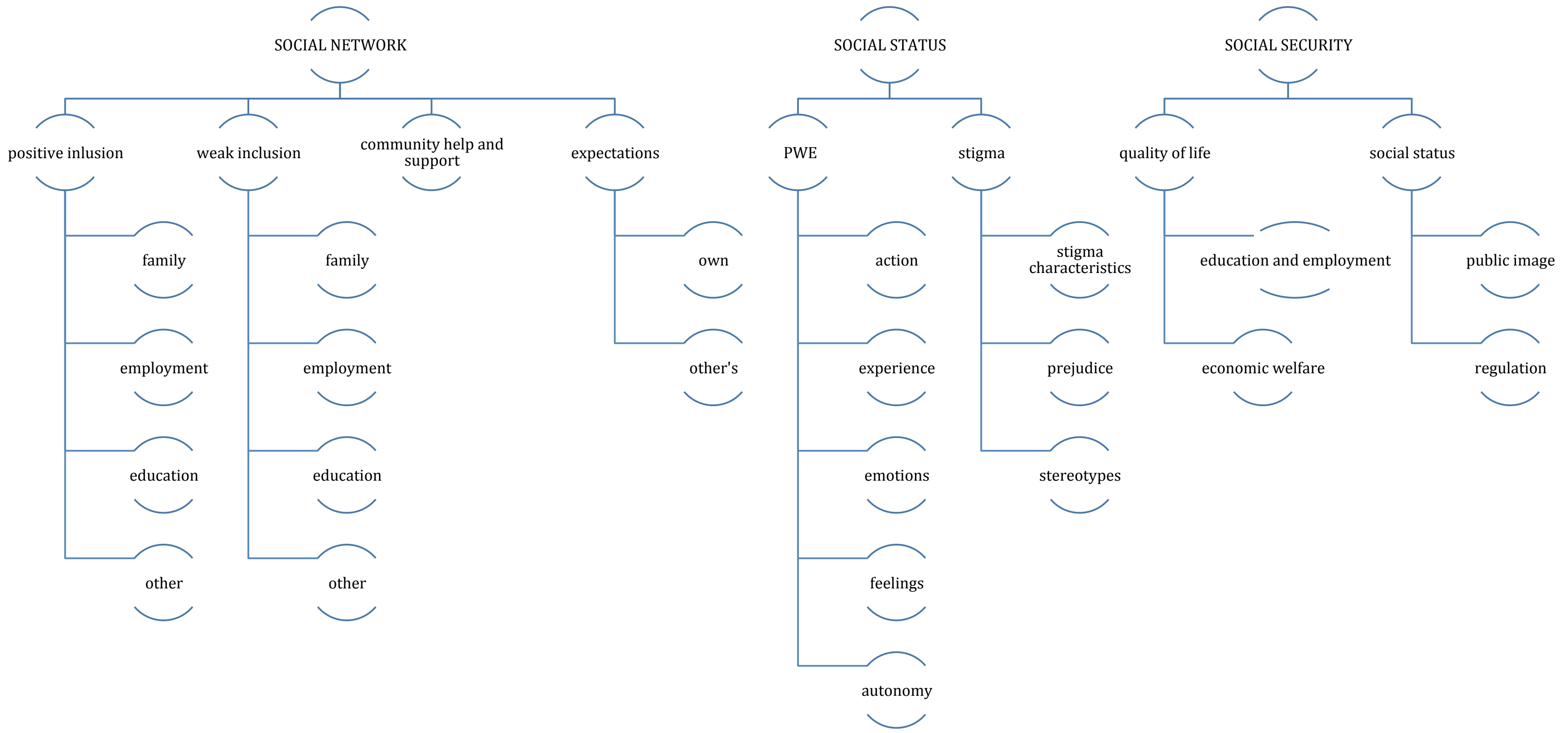

Supplement: Supplementary file 1 — Appendix 1 indicates the placement of categories “Concealing/disclosing epilepsy” and “Epilepsy consequences (disease burden)” within the broader coding frame. Appendix 2 indicates the placement of categories “PWE experience and social network” within the broader coding frame. [file 2018509.f1.pdf]
